# *Mycobacterium fortuitum* skin infection as a complication of anabolic steroids: a rare case report

**DOI:** 10.1308/003588413X13511609955175

**Published:** 2013-01

**Authors:** R Pai, U Parampalli, G Hettiarachchi, I Ahmed

**Affiliations:** Medway NHS Foundation Trust,UK

**Keywords:** *Mycobacterium fortuitum*, Cutaneous abscess, Anabolic steroids

## Abstract

*Mycobacterium fortuitum* is a rare cause of recurrent skin abscesses in an immunocompetent person. We report the case of a 37-year-old man presenting with multiple recurrent non-healing skin abscesses. Culture of the abscess wall yielded growth of *M fortuitum*. In our case, we highlight the association of anabolic steroids with non-tuberculous mycobacterial skin abscesses that fail to resolve despite repeated drainage.

Abscesses requiring incision and drainage present to surgeons frequently. However, it is important to be aware of unusual presentations of abscesses. Those that do not resolve following a surgical procedure or multiply without an obvious cause must be investigated with tissue cultures and wound swabs in order to obtain an accurate diagnosis as inoculation with mycobacteria could be a rare cause.

## Case history

A 37-year-old Caucasian man working as a mechanic presented to the emergency department with a solitary swelling on the right side of his chest wall. He had previously had an incision and drainage of the abscess on the same site while working in the Middle East. He was not diabetic and was immunocompetent. He had injected anabolic steroids in the pectoral muscles for body building in the past. On examination, he had a 5cm × 5cm abscess in the right axilla. It was incised and drained under general anaesthesia and he was discharged home with a course of oral amoxicillin and clavulanate 625mg three times a day for seven days. A routine bacterial culture of the wound showed no significant growth.

**Figure 1:**
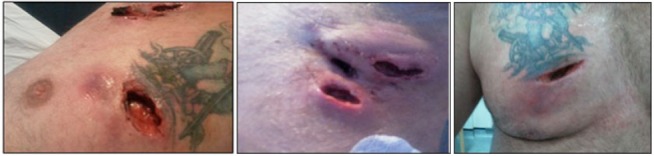
The incised and drained abscesses prior to the administration of antibiotic therapy

The patient presented again a week later with recurrence of the abscess. Further incision and drainage was performed and again the swabs did not yield any bacterial growth. A week later, he presented for a third time with yet another recurrence. The abscesses were erythematous, tender and discharging pus. He underwent further drainage of the abscess cavities. On this occasion, a tissue sample was sent for histology and microbiology analysis. The impression at this time was one of hidradenitis suppurativa (Fig 1).

**Figure 2:**
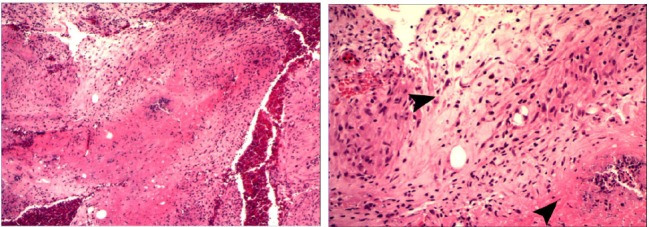
Histology slides: granulomatous inflammation with central necrosis (40× magnification; left) and granuloma in the centre of the picture and caseation in the bottom right (200× magnification; right)

These indurated and multiple abscesses required a fourth round of surgery. The histology was reported as ‘chronic inflammation with multinucleate giant cells along with foci of caseating necrosis surrounded by granulomas’ (Fig 2). There were no acid-fast bacilli demonstrated on special staining. However, the diagnosis was thought to be probable tuberculosis.

**Figure 3:**
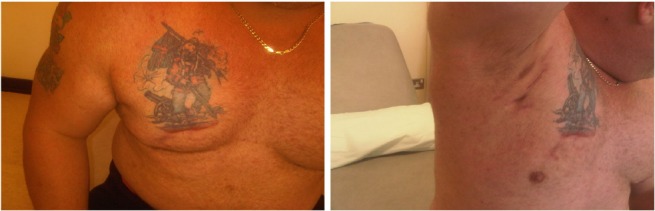
The healed abscesses after surgical debridement and antibiotics therapy

The patient was referred to the respiratory physicians for treatment of tuberculosis. However, the tissue culture isolated a very resistant *Mycobacterium fortuitum*, and so he was treated with ciprofloxacin 750mg twice daily and doxycycline 200mg daily. He was unable to tolerate ciprofloxacin and was switched to a second line quinolone in the form of moxifloxacin 400mg daily and continued on doxycycline 200mg daily. Unfortunately, he developed side effects with the second quinolone and this was also discontinued. The extensive antibiotic resistance pattern meant there were few oral treatment options. Computed tomography of the chest excluded secondary pulmonary involvement. The respiratory physician referred him back for further surgical excision with a view to obtain clear margins. He was reviewed in the surgical clinic few weeks later. The wound had healed completely (Fig 3). Histology revealed only fibrous scar tissue and no evidence of any granulomatous inflammation.

## Discussion

*M fortuitum* is a fast-growing, non-tuberculous mycobacterium that was discovered in the 1930s.^1^ It is a cause of pulmonary, soft tissue and disseminated infections, particularly in immunocompromised patients. It has been most commonly found nosocomially, after contamination of a surgical wound and following prostheses insertion, especially after prosthetic breast implantation^2^ or orthopaedic operations.^3^

*M fortuitum* is usually found in the immunocompromised, for example following chemotherapy^4^ and long-term steroid therapy.^5^ There have been few case reports of *M fortuitum* causing spontaneous skin abscesses in otherwise healthy individuals.^6–8^ Our patient was fit and healthy apart from a history of recreational anabolic steroid use. He was investigated for human immunodeficiency virus and found to be negative.

In our case, we have seen a rare complication of anabolic steroid injection, which may have contributed by both suppressing the immune system and inoculating the organism. Immunological effects of anabolic steroids depend on the type of steroid and the dose administered. Common androgens such as testosterone and nandrolone may focus preferentially on altering immune function by reducing natural killer cell activity and inhibiting the maturation of stem cells into B lymphocytes. At supraphysiological doses, they influence cytokine production directly. All these effects in combination affect immunocompetence although long-term effects are still unproven.^9^

*M fortuitum* is mostly sensitive to amikacin, cefotaxime, gentamicin quinolones and tetracyclines. Although macrolide sensitivity is often detected in vitro, *M fortuitum* contains an inducible resistant gene. Ciprofloxacin and doxycycline are therefore the most common antibiotics used to treat this type of infection. In the majority of cases, dual therapy is recommended. The course of antibiotics should be continued for 4–6 months.^10^ In cases where antibiotic resistance is detected or when the patient is unable to tolerate antibiotics, surgical excision should be considered.

If abscesses are thought to be due to mycobacterial infection, it is important to institute a two-step treatment: incision and drainage of the abscess, and appropriate antimicrobial therapy.^11^ The surgeon operating on the abscess should be careful to remove as much tissue from the site as possible to reduce the burden of infection.

## Conclusions

To our knowledge, this is the first reported case of an association between anabolic steroid injection and *M fortuitum* infection. In this case, surgical excision and prompt antimicrobial treatment achieved a local cure. The key message from our report is that recurrent non-healing skin abscesses should be treated with suspicion for rare organisms, including mycobacterium, and tissue samples should be sent for histology and microbiology.
